# Prognostic models for breast cancer: based on logistics regression and Hybrid Bayesian Network

**DOI:** 10.1186/s12911-023-02224-1

**Published:** 2023-07-13

**Authors:** Fan Su, Jianqian Chao, Pei Liu, Bowen Zhang, Na Zhang, Zongyu Luo, Jiaying Han

**Affiliations:** 1 Department of Epidemiology and Health Statistics, School of Public Health, Southeast University, No. 87 Ding Jia Qiao, Central Gate Street, Gulou District, Nanjing, Jiangsu China; 2 Department of Medical Insurance, School of Public Health, Southeast University, No. 87 Ding Jia Qiao, Central Gate Street, Gulou District, Nanjing, Jiangsu China

**Keywords:** Breast cancer, Prognostic model, Hybrid bayesian network, HER2

## Abstract

**Background:**

To construct two prognostic models to predict survival in breast cancer patients; to compare the efficacy of the two models in the whole group and the advanced human epidermal growth factor receptor-2-positive (HER2+) subgroup of patients; to conclude whether the Hybrid Bayesian Network (HBN) model outperformed the logistics regression (LR) model.

**Methods:**

In this paper, breast cancer patient data were collected from the SEER database. Data processing and analysis were performed using Rstudio 4.2.0, including data preprocessing, model construction and validation. The L_DVBN algorithm in Julia0.4.7 and bnlearn package in R was used to build and evaluate the HBN model. Data with a diagnosis time of 2018(n = 23,384) were distributed randomly as training and testing sets in the ratio of 7:3 using the leave-out method for model construction and internal validation. External validation of the model was done using the dataset of 2019(n = 8128). Finally, the late HER2 + patients(n = 395) was selected for subgroup analysis. Accuracy, calibration and net benefit of clinical decision making were evaluated for both models.

**Results:**

The HBN model showed that seventeen variables were associated with survival outcome, including age, tumor size, site, histologic type, radiotherapy, surgery, chemotherapy, distant metastasis, subtype, clinical stage, ER receptor, PR receptor, clinical grade, race, marital status, tumor laterality, and lymph node. The AUCs for the internal validation of the LR and HBN models were 0.831 and 0.900; The AUCs for the external validation of the LR and HBN models on the whole population were 0.786 and 0.871; the AUCs for the external validation of the two models on the subgroup population were 0.601 and 0.813.

**Conclusion:**

The accuracy, net clinical benefit, and calibration of the HBN model were better than LR model. The predictive efficacy of both models decreased and the difference was greater in advanced HER2 + patients, which means the HBN model had higher robustness and more stable predictive performance in the subgroup.

**Supplementary Information:**

The online version contains supplementary material available at 10.1186/s12911-023-02224-1.

## Introduction

Breast cancer is a malignant tumor that occurs in the glandular epithelial tissue of the breast in women [1]. The latest global cancer burden data released by the World Health Organization’s International Agency for Research on Cancer (IARC) for 2020 shows that there are 2.26 million new breast cancer cases worldwide, surpassing the 2.2 million lung cancer cases. Breast cancer has replaced lung cancer as the number one cancer worldwide [2]. Among breast cancer patients, human epidermal growth factor receptor-2 (HER2)-positive patients account for about 15–20.0%[3, 4]. HER2 + breast cancer is highly aggressive and prone to adverse clinical outcomes with short survival and poor prognosis [5], so it is more critical to predict at an early stage and take therapeutic measures for the possible prognostic outcome of patients such as drug therapy such as pyrrolizumab, trastuzumab or neoadjuvant chemotherapy (NAC) [6, 7]. While most studies currently predict five-year survival in breast cancer, some studies have focused on developing 1-year survival prediction models or Comprehensive Prognostic Index (CPI) for breast cancer patients with multiple comorbidities [8]. Because of the poor prognosis of advanced HER2 + patients, some clinical trials have used 1-year survival as the observed outcome [9]. The follow-up time of breast cancer data with diagnosis in 2019 in the SEER database is less than two years, this study also used 1-year survival as the study outcome, to establish a predictive model to identify people with better and worse prognosis, especially people with a poorer prognosis, and to assist physicians in taking the best interventional treatment for patients promptly.

With the continuous development of machine learning and data mining techniques, more and more researchers have tried to use machine learning models such as Random Forest (RF), Artificial Neural Network (ANN), Decision Tree (DT), and Support Vector Machine (SVM) to build adverse event prediction models. But most of them work as black boxes with a lack of interpretability. Bayesian Network (BN) is a probabilistic graphical model combining probability theory and graph theory, which uses directed acyclic graphs to represent the probabilistic dependencies between nodes [10, 11], intuitively illustrates the interrelationships between variables and can predict the ending variables when some of the nodes are unknown, and has been increasingly applied to various medical fields in recent years, such as building diagnostic and prognostic models for tumor patients [12], constructing risk prediction models for adverse cardiovascular events [13], constructing prediction models for hepatic encephalopathy [14], etc. Traditional Bayesian networks are only used for discrete variables, but many continuous variables exist in practical studies. In addition to the most common equal-width discretization or discretization based on expert experience, a more reasonable method is the minimum description length (MDL) principle discretization. MDL methods trade off goodness-of-fit against model complexity to reduce generalization error. But the MDL method suffers from low sensitivity to discretization edge locations and returns too few discretization intervals for continuous variables [15, 16]. Some researchers have proposed new algorithms based on traditional Bayesian networks, such as the Conditional Gaussian Bayesian network (CGBNs) algorithm [17], which can achieve the simultaneous inclusion of continuous and discrete variables, but it requires that continuous variables cannot be the parent node of discrete variables and continuous variables need to conform to Gaussian distribution. The Learning Discrete Valued Bayesian Networks (L_DVBN) algorithm is a principled Bayesian discretization method for continuous variables in Bayesian networks [18], which combine multi-variable discretization with greedy search [19, 20]. The traditional Bayesian structure learning algorithm needs discretization data, while the proposed discretization algorithm needs to know the network structure. L_ DVBN algorithm can be combined with the K2 structure learning algorithm to simultaneously perform Bayesian network structure learning and discretization of continuous variables. In short, the dataset is initially discretized, and K2 is run to obtain an initial network structure. Then the affected continuous variables are rediscretized every time K2 adds an edge. The resulting discretization policies are used to update the discretized dataset, and the next step of the K2 algorithm is executed. This progress is repeated until the K2 algorithm converges. Research has shown that this method is better than the minimum description length algorithm. Therefore, this study chooses to apply the L_DVBN algorithm to discretize continuous variables and construct a Hybrid Bayesian Network (HBN) prediction model, which can broaden the application of Bayesian networks on continuous variables [19]. The HBN model is compared with the traditional LR model to evaluate the effectiveness comprehensively. In previous studies, prognostic models were found to perform poorly in patients with specific characteristics, such as patients with BRCA1-mutation [21], patients with lympho-vascular invasion [22], HER2 + patients [23], young and old patients [24], etc. In this paper, the two prognostic models constructed were validated separately in advanced HER2 + patients to compare the differences in their efficacy in overall patients and subgroups of patients.

This study followed the “ABCD” criteria for model validation in the comprehensive evaluation system of clinical prediction models [25, 26]; and conducted a thorough evaluation of the efficacy of the constructed LR and HBN models to analyze whether the HBN model based on the L_DVBN algorithm was superior to the traditional LR model and their performance in the whole patients and subgroups, which provided basic ideas for the construction, evaluation, and study of the applicable population of breast cancer prognostic models in the future.

## Related work

Past studies commonly used Logistic Regression (LR), Cox regression, and the Kaplan-Meier test for survival prediction of tumor patients [27–29]. The development of big medical data and electronic medical record systems makes machine learning models applied to breast cancer patients’ diagnosis, recurrence, lymph node metastasis, and survival outcome prediction [30].

Research has built a diagnosis model for breast cancer using feature selection procedures to select the most valuable feature, 13 classification algorithms including Gaussian Naive Bayes and Gaussian Naive Bayes. Model that used logistics regression feature selection procedure and Multilayer Perceptron (MLP) classifier performed best [31]. Jose et al. [32]used decision trees based on control of induction by sample division method to select prognosis factors for different time intervals during the follow-up time of the patients. Then input prognostic factors into specific topologies of neural network systems to obtain good accuracy of the classification probability of breast cancer patients. Kim et al. [33] constructed a prognostic model based on support vector machine (SVM) for predicting breast cancer recurrence within five years after breast cancer surgery in the Korean population. Compared with well-known models(St. Gallen guidelines, NPI, and Adjuvant! Online), SVM has a high degree of differentiation. Gaosen et al. [34] 10 machine learning models, including naive Bayesian network to predict sentinel lymph node for evaluating the preoperative diagnostic value of ultrasound signs of breast cancer lesions for sentinel lymph node (SLN) metastasis. The study also used SHapley Additive exPlanation (SHAP) to visualize the diagnostic process of the ML model. Wang et al. [35] used logistics regression and C5 Decision Trees(DT) based on the Synthetic minority oversampling technique (SMOTE) and Synthetic minority oversampling technique(PSO)to predict the 5-year survival rate of patients with breast cancer. SMOTE algorithm was used to handle unbalanced data; PSO algorithm was used for feature selection. Durson et al. constructed ANN, DT, and logistics regression. They found that the DT is the best predictor with 93.6% accuracy on the holdout sample. Artificial neural networks came out to be the second with 91.2% accuracy. They are all better than logistics regression(89.2% accuracy).

Considering many models, such as artificial neural network work as black boxes with a lack of explainability, Some studies try to combine Bayesian network with these “black boxes” models. Nurduman et al. combined Convolutional Neural Networks and Bayesian Networks with IR thermal images to achieve good diagnostic accuracy from a dataset of images and data [36]. The accuracy, accuracy, sensitivity, and specificity of the model are all above 90%. Jong et al. developed a hybrid Bayesian network model to predict breast cancer prognosis. By inserting the confidence value of the neural network as a node into the hybrid Bayesian network model, the hybrid Bayesian network is both highly accurate and interpretable. The AUC of the HBN model is 0.935, higher than that of the neural network and Bayesian network [37].

There are also some research innovations in data sources, missing value interpolation methods, and discretization when building Bayesian network. There have been research developed Bayesian networks to integrate clinical and microarray data. The results show that using two types of data together outperforms the indices based on clinical data. The AUC of the model is higher than 0.8, indicating that the Bayesian network model can reasonably predict whether the prognosis of patients is good or poor [38]. In addition, Some studies have used Bayesian network to input missing values of discrete fields in combination with Tensor factorization to improve interpolation accuracy [39]. Friedman et al. proposed discretization of continuous variables based on MDL principle when building Bayesian network. They use the MDL principle to select the threshold values while learning the Bayesian network structure for discretization. This method balances the completeness of the dispersion and Goodness of fit of the structure. Friedman et al. proposed discretization of continuous variables based on MDL principle when building Bayesian network. They use the MDL principle to select the threshold values while learning the Bayesian network structure for discretization. This method balances the completeness of the dispersion and Goodness of fit of the structure (16).

This study combines Bayesian network with L_ DVBN algorithm is combined to build a hybrid Bayesian network. Pass L_ DVBN algorithm can better discretize the variables on the discretization variable Markov blanket and then improve the accuracy of the prediction model.

## Method

### Study population

The data of breast cancer patients in this article were obtained from the incidence data in the Surveillance, Epidemiology, and End Results (SEER) database: SEER Research Plus Data, 8 Registries, Nov 2021 Sub (1975–2019) and SEER Research Plus Data, 12 Registries, Nov 2021 Sub (1992–2019), The SEER data files were requested through the SEER web site (http://www.seer.cancer.gov). Access to the data in this study was obtained by signing the SEER data study protocol and therefore did not require ethics committee approval or informed consent. Table 1 shows data inclusion criteria and exclusion criteria.

**Table 1:** Data inclusion and exclusion criteria

Inclusion criteria	Exclusion criteria
• Female	• Died due to other diseases
• Diagnosed in 2018 or 2019	• With death certificate only
• ICD-O-3 disease codes 8500/8507/8520–8524/8530/8540–8543	• Lost to follow-up
• ICD-O-3 behavior codes 3	• Unknown cause of death
• Only one primary site	
• Positive pathological diagnosis	

### Measurements

Referring to AJCC guidelines, CS tumor information collection system, and related literature, 17 study variables were extracted in SEER: Age, Stage, Tumor size, Primary site, Race, Marital status, Grade, Histologic Type ICD-O-3, ER Status, PR Status, Radiation, Laterality, CS lymph nodes, RX Summ–Surg Prim Site, Chemotherapy recode, EOD Mets, and Subtype, with the two variables Age and Tumor size being continuous variables and the rest being categorical variables.

The 5-year survival of breast cancer patients is an important indicator to evaluate the prognostic effect. However, the prognosis of the advanced HER2 + subgroup is poor. The existing studies for this subgroup have also used 1-year survival to evaluate the prognostic effect of a specific treatment. In this paper, the 18-months survival of breast cancer patients is used as an outcome indicator to evaluate the effect of the prediction model. The original variable “Survival Months” more than or equal to 18 months is considered as “survival”, and less than 18 months is considered as “death”.

### Features selection

Univariate analysis was conducted to identify significant differences between the survival and death groups using univariate logistics regression, chi-square test, or Fisher’s exact test. If variables were not significant in univariate analysis, other features were additionally selected based on expert advice and published literature. Variables that did not conform to normal distribution were expressed as median (M) and quartiles (P25, P75), and univariate logistics regression was used for comparison between groups; count data were expressed as composition ratio, and the chi-square test or Fisher’s exact test was used for comparison between groups.

### Data pre-processing

Data pre-processing was performed using Rstudio 4.2.0. The main steps included variable recoding, visualization of missing data using the VIM package, interpolation of missing data by multiple interpolations using the mice package, and sample balancing using the DMwR package. The final sample size was 31,131.

### Model development

A multivariate LR prognostic model was constructed using stepwise (forward-backward method) with α_in = 0.10 and α_out = 0.15. A hybrid Bayesian network(HBN) model was constructed using the Learning Discrete Valued Bayesian Networks (L_DVBN) algorithm proposed by Chen et al. [19].

### Model effect evaluation

The internal validation of the model was performed using the leave-out method, dividing the sample into the training set and testing set according to 7:3, using the training set to construct two prediction models, and using the testing set to internally validate the constructed model, selecting the incidence data from the SEER database: SEER Research Plus Data, 12 Registries, Nov 2021 Sub (1992–2019) with a diagnosis time of 2019 and in registries different from the internal validation data for external validation. The external validation sample size was 20,320. The pROC package was used to do the Area Under Curve (AUC) of the Receiver Operating Characteristic Curve (ROC) to evaluate the accuracy of the model, the rms package was used to plot the calibration curve to evaluate the calibration of the model, and the dca function (from the website: https://www.mskcc.org/departments/epidemiology-biostatistics/biostatistics/) was used to plot the DCA decision curve to evaluate the net benefit of the model for clinical decision making, to compare the efficacy of the two models comprehensively.

### Subgroup analysis

Human epidermal growth factor receptor 2 (HER2)-positive breast cancer is a common subtype of breast cancer with a worse prognosis than HER2- patients. Therefore, the constructed multivariable logistic regression prediction model and the HBN model were externally validated in the advanced HER2 + patients with a sample size of n = 1390, and the results of the external validation were compared with those of the overall patients to evaluate the predictive effect of the two prediction models in this subgroup of the patients.

## Results

### Basic characteristics of the research object

Based on inclusion and exclusion criteria, Data from SEER Research Plus Data, 8 Registries, Nov 2021 Sub (1975–2019) with a diagnosis time of 2018 (n = 15,053) were used for model construction and internal validation. Data from SEER Research Plus Data, 12 Registries, Nov 2021 Sub (1992–2019) with a diagnosis time of 2019 (n = 5871) were used for external validation of the model. Due to the data imbalance, the 2018 data were balanced using the SMOTE algorithm of the DMwR package so that survival and death cases accounted for 50% of the total. The balanced data were randomly allocated as training set (n = 16,370) and testing set (n = 7014) with a ratio of 7:3. External validation was done using data from 2019 after sample balancing by SMOTE algorithm (n = 8128). Cases with T3 or T4 and positive HER2 receptor (n = 395) were screened in the 2019 data for subgroup analysis. The datasets used were summarized in Table 2.

**Table 2:** Summary of datasets

Dataset	Sample Size		
	All(*N*%)	Dead(*N*%)	Survival(*N*%)
Original data	15,053	11,207 (74.45%)	3846 (25.55%)
External validation data	5871	3839 (65.39%)	2032 (34.61%)
Subgroup analysis data	395	276 (69.87%)	119 (30.13%)

### Distribution of survival outcomes in populations with different characteristics

Univariate analyses of the relationship between baseline patient characteristics and survival outcomes were performed. The age of patients in the survival group was 61.0 [51.0, 70.0] years and 60.0 [51.0;66.0]years in the death group, and the difference in age between the two groups was not statistically significant, i.e., $p>0.05$. The tumor size was 17.0 [10.0;28.0] mm in the survival group and 23.0 [15.0;36.0] mm in the death group, and the difference between the two groups was statistically significant, i.e., $p<0.001$. The differences in the distribution of benign and malignant pulmonary nodules among different ages, tumor size, pathological types, radiotherapy, surgery, chemotherapy, distant metastasis status, subtype, clinical stage, ER receptor, PR receptor, clinical grade, primary site, race, marital status, laterality, and lymph node metastasis status were statistically significant$(p<0.05)$, as shown in Table 3.

**Table 3:** Distribution of survival outcomes in populations with different features

Characteristics	Outcome		*p* value
	Survival N = 29,230(%)	Dead N = 29,230(%)	
Age (year)	61.0 [51.0;70.0]	60.0 [51.0;66.0]	<0.001^***^
Tumor Size (mm)	17.0 [10.0;28.0]	23.0 [15.0;36.0]	0.000^***^
Radiation			<0.001^***^
Beam radiation	18,072 (61.8%)	14,449 (49.4%)	
Radioactive implants	432 (1.48%)	451 (1.54%)	
Others	10,726 (36.7%)	14,330 (49.0%)	
Surg			0.000^***^
No	2119 (7.25%)	9845 (33.7%)	
Yes	27,111 (92.8%)	19,385 (66.3%)	
Chemotherapy			0.000^***^
No	18,207 (62.3%)	13,326 (45.6%)	
Yes	11,023 (37.7%)	15,904 (54.4%)	
Mets			0.000^***^
No distant metastasis	27,891 (95.4%)	18,461 (63.2%)	
No evidence of distant mets	43 (0.15%)	47 (0.16%)	
Distant lymph node(s)	129 (0.44%)	759 (2.60%)	
Others	1167 (3.99%)	9963 (34.1%)	
Subtype			0.000^***^
HR-/HER2-	2856 (9.77%)	5186 (17.7%)	
HR-/HER2+	1119 (3.83%)	1575 (5.39%)	
HR+/HER2-	22,302 (76.3%)	17,406 (59.5%)	
HR+/HER2+	2953 (10.1%)	5063 (17.3%)	
Stage			0.000^***^
1 A	16,899 (57.8%)	10,961 (37.5%)	
1B	4841 (16.6%)	3438 (11.8%)	
2 A	2849 (9.75%)	1962 (6.71%)	
2B	1446 (4.95%)	1012 (3.46%)	
3 A	792 (2.71%)	448 (1.53%)	
3B	727 (2.49%)	436 (1.49%)	
3 C	380 (1.30%)	291 (1.00%)	
4	1296 (4.43%)	10,682 (36.5%)	
ER			<0.001^***^
Negative	4283 (14.7%)	7759 (26.5%)	
Positive	24,947 (85.3%)	21,471 (73.5%)	
PR			<0.001^***^
Negative	7288 (24.9%)	10,934 (37.4%)	
Positive	21,942 (75.1%)	18,296 (62.6%)	
Histologic			0.000^***^
8500	24,910 (85.2%)	20,488 (70.1%)	
8507	97 (0.33%)	370 (1.27%)	
8520 or 8521	2992 (10.2%)	4080 (14.0%)	
8522	1041 (3.56%)	2777 (9.50%)	
8523 or 8524	135 (0.46%)	219 (0.75%)	
8530	31 (0.11%)	1238 (4.24%)	
Others	24 (0.08%)	58 (0.20%)	
Grade			0.000^***^
1	6392 (21.9%)	4349 (14.9%)	
2	10,701 (36.6%)	7738 (26.5%)	
3	5282 (18.1%)	4281 (14.6%)	
4	6855 (23.5%)	12,862 (44.0%)	
Site			0.000^***^
C501	1309 (4.48%)	1727 (5.91%)	
C502	3985 (13.6%)	2916 (9.98%)	
C503	1629 (5.57%)	1226 (4.19%)	
C504	10,291 (35.2%)	10,142 (34.7%)	
C505	2398 (8.20%)	1420 (4.86%)	
C508	6938 (23.7%)	6190 (21.2%)	
C509	2680 (9.17%)	5609 (19.2%)	
Race			0.000^***^
White	22,030 (75.4%)	17,832 (61.0%)	
Black	2719 (9.30%)	6324 (21.6%)	
Others	4481 (15.3%)	5074 (17.4%)	
Marital			0.000^***^
Married	18,114 (62.0%)	14,102 (48.2%)	
Divorced	3115 (10.7%)	3195 (10.9%)	
Separated	209 (0.72%)	664 (2.27%)	
Single	4428 (15.1%)	8093 (27.7%)	
Unmarried or Domestic Partner	302 (1.03%)	340 (1.16%)	
Widowed	3062 (10.5%)	2836 (9.70%)	
Laterality			<0.001^***^
Left - origin of primary	14,886 (50.9%)	13,596 (46.5%)	
Right - origin of primary	14,344 (49.1%)	15,634 (53.5%)	
Node			$<$0.001^***^
0	6995 (23.9%)	5838 (20.0%)	
1	13,616 (46.6%)	8923 (30.5%)	
2	1056 (3.6%)	710 (2.4%)	
3	1967 (6.7%)	5946 (20.3%)	
4	4418 (15.1)	4123 (14.1%)	
5	204 (0.7%)	119 (0.4%)	
6	112 (0.4%)	136 (0.5%)	
7	199 (0.7%)	921 (3.2%)	
8	14 (0.0%)	30 (0.1%)	
9	160 (0.5%)	174 (0.6%)	
10	162 (0.6%)	757 (2.6%)	
11	156 (0.5%)	1083 (3.7%)	
12	171 (0.6%)	470 (1.6%)	

^*^means P $\le$ 0.05; ^**^means P $\le$ 0.01; ^***^means P $\le$ 0.001

Surg, Primary Site Surgery; Nodes, Lymph Node; Mets, Distant Metastasis; ER, Estrogen Receptor; PR, Progesterone Receptor; Histologic, Histologic Type; Site, Primary Site

### LR model and HBN model construction and effect evaluation

#### LR model construction

A prognostic model was constructed using the training set with the outcome variables (0 = death, 1 = survival). Based on expert experience and previous studies, it was concluded that age also affects patient prognosis, so variables statistically significant in the univariate analysis and age were used as independent variables in stepwise logistic regression. According to α_in = 0.10 and α_out = 0.15 criteria, pathological type, radiotherapy, surgery, chemotherapy, distant metastasis status, subtype, clinical stage, ER receptor, PR receptor, clinical grade, primary site, race, marital status, tumor laterality, lymph node metastasis was statistically significant and included in the final model (Table 4).

**Table 4:** LR model for predicting survival outcomes

Characteristics	$\beta$	${s}_{\stackrel{-}{x}}$	*Wald* ${\chi }^{2}$	*OR* [95%CI]	*p* value
Age	-0.006	0.001	-5.540	0.994 [0.992, 0.996]	< 0.001^***^
Tumor Size (mm)	0.001	0.001	1.665	1.001 [1.000, 1.003]	$<$0.001^***^
Radiation					
Beam radiation				1.000	
Radioactive implants	0.639	0.097	6.565	1.895 [1.565,2.292]	$<$0.001^***^
Others	0.105	0.026	4.078	1.110 [1.056,1.168]	$<$0.001^***^
Surg					
No				1.000	
Yes	-0.881	0.039	-22.842	0.414 [0.384, 0.447]	$<$0.001^***^
Chemotherapy					
No				1.000	
Yes	0.207	0.030	6.974	1.230 [1.161, 1.304]	$<$0.001^***^
Nodes					
0				1.000	
1	-0.127	0.032	-3.901	0.881 [0.827, 0.939]	< 0.001^***^
2	0.059	0.072	0.818	1.061 [0.920, 1.221]	0.414
3	0.371	0.048	7.735	1.449 [1.319, 1.592]	< 0.001^***^
4	0.296	0.042	7.047	1.345 [1.239, 1.461]	< 0.001^***^
5	-0.238	0.164	-1.452	0.788 [0.569, 1.082]	0.146
6	0.790	0.183	4.327	2.203 [1.539, 3.151]	< 0.001^***^
7	1.025	0.113	9.050	2.787 [2.238, 3.490]	< 0.001^***^
8	-0.424	0.482	-0.880	0.655 [0.257, 1.707]	0.379
9	0.001	0.180	0.003	1.001 [0.703, 1.425]	0.997
10	0.771	0.125	6.183	2.161 [1.698, 2.769]	< 0.001^***^
11	1.240	0.118	10.552	3.457 [2.756, 4.371]	< 0.001^***^
12	0.573	0.135	4.237	1.773 [1.363, 2.316]	< 0.001^***^
Mets					
No distant metastasis				1.000	
No evidence of distant mets	0.618	0.268	2.309	1.855 [1.094, 3.137]	0.021^*^
Distant lymph node(s)	1.144	0.130	8.818	3.139 [2.447, 4.071]	< 0.001^***^
Others	1.413	0.044	31.957	4.110 [3.770, 4.484]	< 0.001^***^
Subtype					
HR-/HER2-				1.000	
h-/HER2+	-0.244	0.064	-3.804	0.783 [0.690, 0.888]	< 0.001^***^
HR+/HER2-	-0.428	0.048	-8.895	0.652 [0.593, 0.716]	< 0.001^***^
HR+/HER2+	-0.003	0.056	-0.050	0.997 [0.893, 1.114]	0.96
Stage					
1 A				1.000	
1B	-0.323	0.039	-8.227	0.724 [0.670, 0.782]	< 0.001^***^
2 A	-0.497	0.049	-10.203	0.608 [0.553, 0.669]	< 0.001^***^
2B	-0.698	0.065	-10.786	0.497 [0.438, 0.565]	< 0.001^***^
3 A	-0.941	0.087	-10.851	0.390 [0.329, 0.462]	< 0.001^***^
3B	-1.139	0.091	-12.552	0.320 [0.268, 0.382]	< 0.001^***^
3 C	-1.292	0.116	-11.121	0.275 [0.218, 0.345]	< 0.001^***^
4	1.192	0.046	25.926	3.294 [3.012, 3.606]	< 0.001^***^
ER					
Negative				1.000	
Positive	-0.395	0.042	-9.476	0.674 [0.621, 0.731]	$<$0.001^***^
PR					
Negative				1.000	
Positive	-0.258	0.032	-8.147	0.773 [0.726, 0.822]	$<$0.001^***^
Histologic					
8500				1.000	
8507	1.725	0.152	11.381	5.614 [4.195, 7.607]	$<$0.001^***^
8520 or 8521	0.644	0.038	17.015	1.904 [1.768, 2.051]	$<$0.001^***^
8522	1.110	0.053	21.113	3.036 [2.739, 3.366]	$<$0.001^***^
8523 or 8524	1.050	0.147	7.131	2.858 [2.143, 3.820]	$<$0.001^***^
8530	2.872	0.218	13.148	17.675 [11.795, 27.880]	$<$0.001^***^
Others	1.186	0.303	3.916	3.273 [1.833, 6.047]	$<$0.001^***^
Grade					
1				1.000	
2	-0.094	0.036	-2.581	0.911 [0.848, 0.978]	0.01
3	-0.043	0.045	-0.957	0.957 [0.876, 1.047]	0.339
4	-0.028	0.043	-0.659	0.972 [0.894, 1.057]	0.51
Site					
C501				1.000	
C502	-0.379	0.064	-5.905	0.685 [0.604, 0.776]	< 0.001^***^
C503	-0.349	0.077	-4.561	0.705 [0.607, 0.819]	< 0.001^***^
C504	-0.217	0.057	-3.785	0.805 [0.719, 0.901]	< 0.001^***^
C505	-0.572	0.072	-7.898	0.564 [0.489, 0.650]	< 0.001^***^
C508	-0.309	0.059	-5.208	0.734 [0.653, 0.825]	< 0.001^***^
C509	0.101	0.064	1.591	1.107 [0.977, 1.254]	0.112
Race					
White				1.000	
Black	0.620	0.036	17.003	1.859 [1.731, 1.997]	$<$0.001^***^
Others	0.246	0.033	7.368	1.279 [1.198, 1.365]	$<$0.001^***^
Marital					
Married				1.000	
Divorced	0.166	0.040	4.169	1.181 [1.092, 1.276]	$<$0.001^***^
Separated	0.685	0.113	6.053	1.985 [1.592, 2.483]	$<$0.001^***^
Single	0.427	0.032	13.327	1.532 [1.439, 1.631]	$<$0.001^***^
Unmarried or Domestic Partner	0.203	0.119	1.704	1.224 [0.969, 1.545]	0.088
Widowed	0.042	0.044	0.942	1.043 [0.956, 1.137]	0.346
Laterality					
Left - origin of primary				1.000	
Right - origin of primary	0.089	0.024	3.674	1.094 [1.043, 1.147]	$<$0.001^***^

^*^ means P $\le$ 0.05; ^**^ means P $\le$ 0.01; ^***^ means P $\le$ 0.001

Surg, Primary Site Surgery; Nodes, Lymph Node; Mets, Distant Metastasis; ER, Estrogen Receptor; PR, Progesterone Receptor; Histologic, Histologic Type; Site, Primary Site

#### HBN model construction

**Fig. 1:**
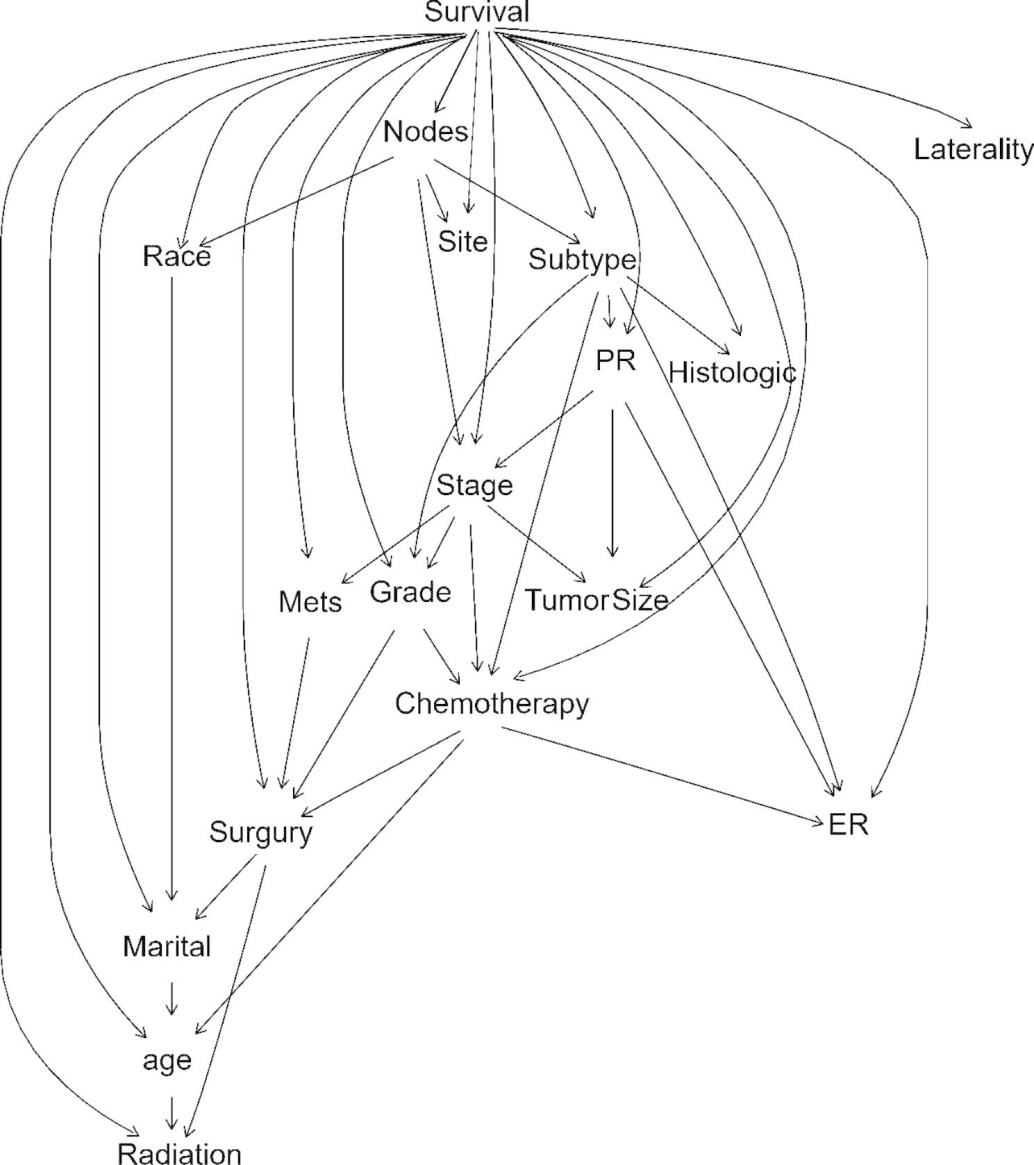
Structure of HBN model

Seventeen significant variables for univariate analysis were included in the HBN model. The L_DVBN algorithm was implemented using Julia 0.4.7 software, and the bnlearn package in Rstudio was used for structure and parameter learning of the HBN model. Structure learning uses the forbidden search method, and parameter learning uses Bayesian estimation. In the BN model, the node from which the arrow emanates is called the parent node, and the node to which the arrow points is called the child node. When the Markov blanket of a node is given, i.e., the values of the parent, child, and child’s parent of that node are given, the node is independent of all other nodes. Based on the above properties, the determination of survival of breast cancer patients is closely related to age, tumor size, subtype, primary site, surgery, radiotherapy, chemotherapy, PR receptor, ER receptor, pathological type, tumor grade and stage, and indirectly or conditionally independent of factors such as laterality, and there is a correlation between the factors (Fig. 1). Strength of arcs between outcome and other variables can be calculated by “arc.strength” function in “bnlearn” package.

#### Evaluation of the effect of LR model and HBN model

**Fig. 2:**
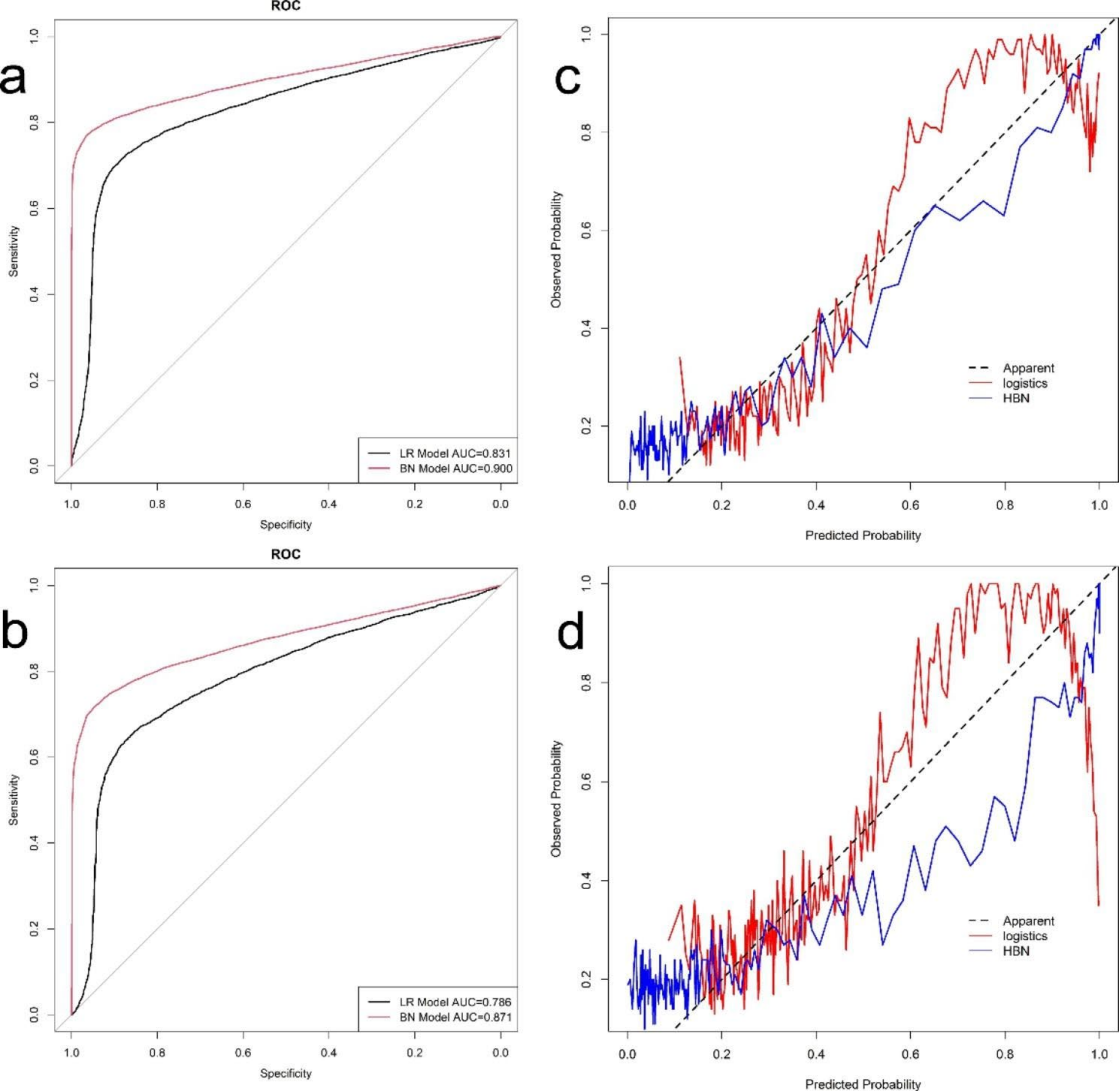
Comparison of ROC curves and calibration curves of LR model and HBN model **a** ROC curve for internal validation of LR model and HBN model, **b** calibration curve for internal validation of LR model and HBN model, **c** ROC curve for external validation of LR model and HBN model, **d** calibration curve for external validation of LR model and HBN model

**Fig. 3:**
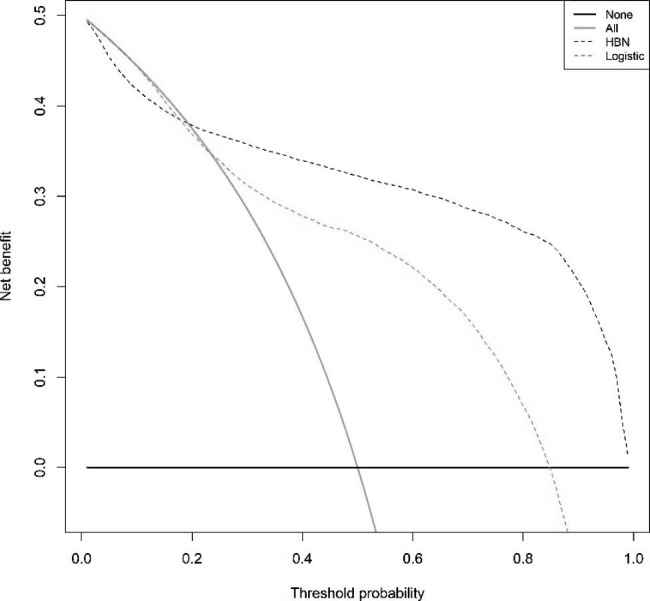
DCA for external validation of LR model and HBN model The abscissa is the threshold probability, and the ordinate is the net benefit rate. None: overall death occurred in no patients, with a net benefit of zero; All: all patients will have overall death at a specific threshold probability; HBN: the net benefit when using the HBN model as a basis for decision; logistics: the net benefit when using the LR model as a basis for decision

The model’s predictive performance is evaluated using the testing set for internal validation, and the ROC curve is plotted to evaluate the model’s accuracy. The AUC (specificity, sensitivity) of the LR model and the HBN model on the testing set were 0.831(0.884,0.715) and 0.900(0.963,0.772), respectively (Supplementary Figs. 1–2, Additional File 1), and the accuracy of the HBN model was slightly higher than that of the LR model. The difference is statistically significant using the Delong test for the two ROC curves, P < 0.001. The calibration curves were drawn to evaluate the calibration of the models The calibration curves showed that the errors between both model’s predicted and actual values were minor and had higher accuracy The calibration curve of the HBN model was better than that of the LR model (Supplementary Figs. 3–4, Additional File 1). We performed external validation of the constructed models and evaluated the efficacy of the models on external data by drawing ROC, calibration, and DCA clinical decision curves. The AUC (specificity, sensitivity) of the LR model and HBN model on 2019 data were (0.876,0.637)0.786 and (0.948,0.717)0.871, respectively (Supplementary Figs. 5–6, Additional File 1), P < 0.001, the differences between the two ROC curves were statistically significant, and the accuracy of the HBN model was still higher than that of the LR model. The external validation calibration curves (Supplementary Figs. 7–8, Additional File 1) were plotted The survival of confirmed patients in 2019 predicted by the model can be obtained from the graphs with a high agreement with the actual observed values. The external validation DCA curves of both models are shown in Supplementary Figs. 9–10, Additional File 1, which show that the net benefit of clinical decision-making according to the prediction model is higher than that of all-treatment or no-treatment, indicating that the model has a higher practical use in clinical decision making and can also produce a better net clinical benefit if applied to other breast cancer populations. The net benefit of the HBN model was higher than that of the LR model. A comparison of the ROC, calibration, and DCA decision curves for the internal and external validation of the two models is shown in Figs. 2 and 3. The accuracy, sensitivity, specificity, net clinical benefit, and calibration of the internal validation of the HBN model were all the better than those of the logistic regression model, except for the calibration of the external validation, which was slightly lower than that of the logistic regression model. The HBN model performed better.

### Subgroup analysis of advanced HER2 + patients

**Fig. 4:**
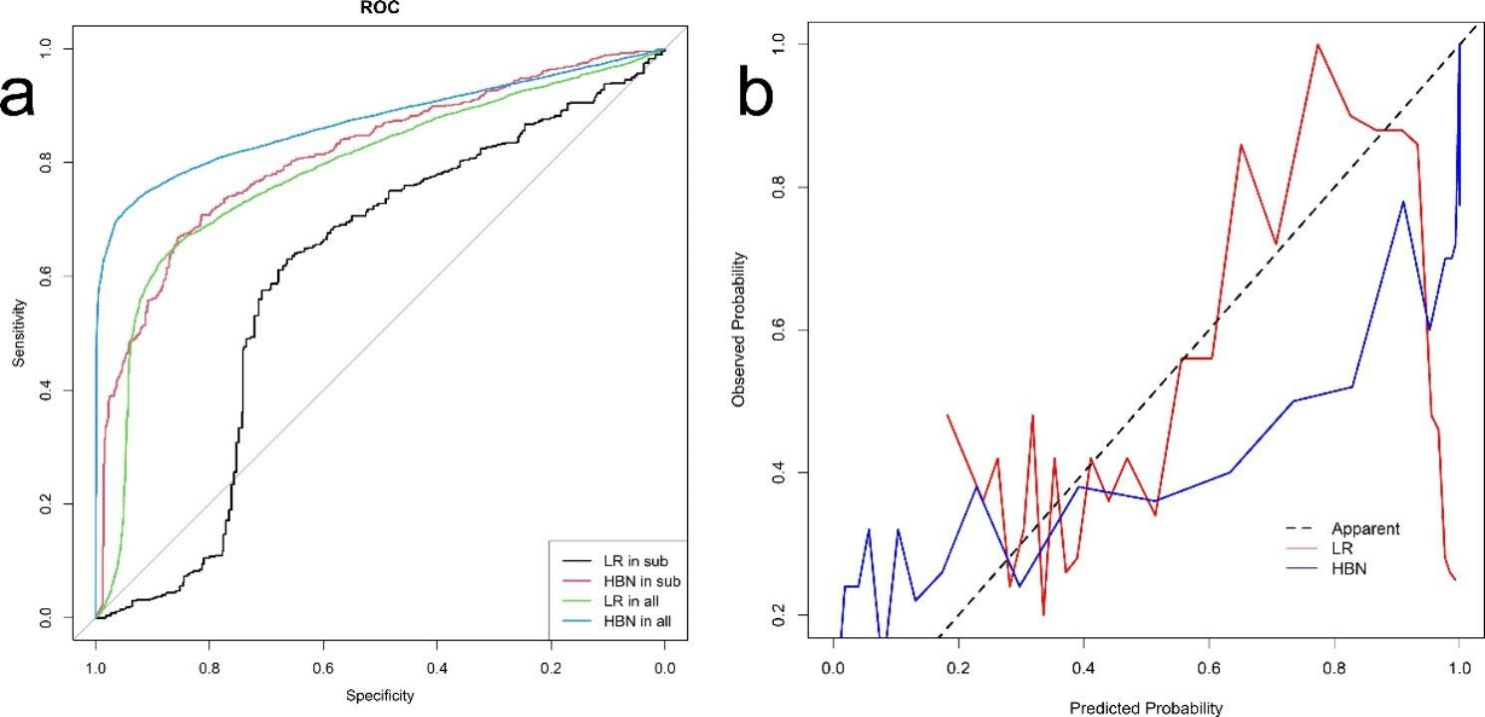
ROC curve and DCA for external validation of LR and HBN model in subgroup **a** HBN in sub: ROC curve for HBN model in the subgroup patients; LR in sub: ROC curve for LR model in the subgroup patients; LR in all: ROC curve for LR model in the overall patients; HBN in all: ROC curve for HBN model in the overall patients, **b** Calibration curve for external validation of LR and HBN model in subgroup

**Fig. 5:**
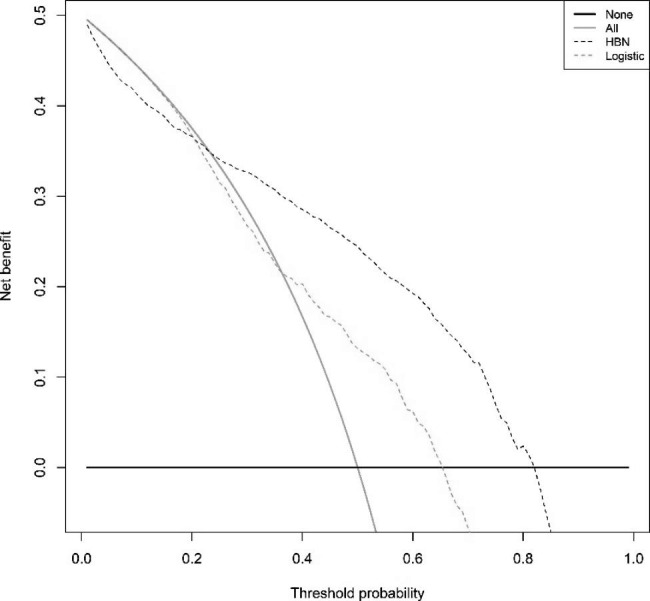
DCA for external validation of LR and HBN model in subgroup The abscissa is the threshold probability, and the ordinate is the net benefit rate. None: overall death occurred in no patients, with a net benefit of zero; All: all patients will have overall death at a specific threshold probability; logistics: the net benefit when using the LR model as a basis for decision; HBN: the net benefit when using the HBN model as a basis for decision

The LR model and the HBN model were externally validated in advanced HER2 + patients to compare the predictive effect of the two prognostic models in this subgroup of patients. In addition, to compare whether there is a difference in the predictive efficacy between the overall breast cancer patients and the subgroup of patients defined in this study. The results of the subgroup analysis are shown in Figs. 4 and 5. the AUC (sensitivity, specificity) of the LR model and HBN model validated in the overall patients and subgroup were 0.786(0.876,0.637), 0.871(0.948,0.717), 0.601(0.663,0.630), 0.813 (0.855,0.669). Besides, the differentiation index of the two models in overall and subgroup patients is summarized in Table 5. The results found that the HBN model was significantly more effective than the logistic regression prediction model in this subgroup of the population in terms of accuracy, calibration, and net clinical benefit. However, the predictive efficacy of either model decreased in advanced HER2 + patients, i.e., accuracy, calibration, and net clinical decision benefit WAS inferior to survival prediction in the overall breast cancer patient population. However, comparing Figs. 2, 3 and 4, it can be observed that the difference in the efficacy of the two models is more significant when predicting advanced HER2 + patients than overall breast cancer patients. Therefore, the HBN model has a higher robustness and a more stable predictive performance in the subgroup population.

**Table 5:** Differentiation index of LR and HBN model

	Model	ACC	TPR	TNR	PPV	NPV
Internal Validation	LR	0.80	0.71	0.88	0.87	0.75
	HBN	0.87	0.77	0.96	0.95	0.81
External Validation	LR	0.76	0.64	0.88	0.84	0.71
	HBN	0.83	0.72	0.95	0.93	0.77
Subgroup Analysis	LR	0.65	0.63	0.66	0.65	0.64
	HBN	0.76	0.86	0.67	0.82	0.72

LR, Logistic regression; HBN, Hybrid bayesian network; ACC, Accuracy; TPR, True positive rate; TNR, True negative rate; PPV, Positive predictive value; NPV, Negative predictive value

## Discussion

In this study, we combined information on the demographic characteristics of breast cancer patients, stage and grade of disease, and treatment history to construct the LR model and HBN model to predict the 18-month survival rate of breast cancer patients and HBN + advanced patients. There were 18 nodes in the model related to survival status (Age, Stage, Tumor size, Primary site, Race, Marital status, Grade, Histologic Type ICD-O-3, ER Status, PR Status, Radiation Laterality, CS lymph nodes, RX Primary Site, Chemotherapy, EOD Mets, Subtype). The HBN model had better predictive accuracy, calibration, and net benefit of clinical decision than the multivariable LR model in both the overall and subgroup, with better predictive performance. As in previous studies, factors such as race, stage, subtype, ER receptor, PR receptor, and lymph node metastasis affect long-term survival [40–42] These relationships are reflected in the Bayesian network model in the form of an arc between two nodes.

Clinical decision analysis can reflect the net benefits of making decisions based on the model results. The decision curves of the two models constructed in this study are superior to those of “treat all” and “treat none”. Regardless of the ratio between the benefits of successfully identifying survival patients (true positive) and the losses of incorrectly identifying survival patients (false positive), using predictive results to determine whether to intervene always brings net benefits. Therefore, appropriate interventions can be selected based on the patient’s predicted results. For example, active individualized treatment should be implemented for patients with a high likelihood of 18-month survival based on their disease status; For patients with a low probability of survival, if they have not received surgery, radiotherapy, or chemotherapy, timely intervention should also be taken; If the patient has already intervened in treatment, they can choose palliative treatment or tranquilization therapy, which can avoid not only unnecessary pain caused by treatment, but also avoid excessive medical treatment and waste of medical resources. In practice, it is more important to identify as many patients with poorer prognoses as possible and intervene in their treatment on time. In this study, The specificity of the model is relatively high, so it can accurately identify patients with poor prognoses.

As in previous studies, factors such as race, stage, subtype, ER receptor, PR receptor, and lymph node metastasis affect long-term survival [38–40], and these relationships are reflected in the Bayesian network model in the form of an arc between two nodes. Compared with the traditional logistics model, the Bayesian network has several advantages. First, there is an association between risk factors, clinical characteristics, and disease. In logistic regression, the variables must be independent, and a linear relationship between the independent and dependent variables is required [43], which sometimes differs from the actual situation. Nevertheless Bayesian networks have no strict requirements for statistical assumptions and can reveal multifactorial and multilevel interactions. Secondly, Bayesian networks show these relationships graphically, which is more concise and clear than correlation coefficients, multivariate correlation line graphs, etc. Traditional Bayesian networks are only applicable to discrete variables; if there are continuous variables, the common practice is to discretize them before modeling based on medical reference values or algorithms, which makes model construction simpler but does not take into account the interplay between discretization and modeling processes, and the original information is easily lost. The hybrid Bayesian network based on the L_DVBN algorithm combines the discretization process of continuous variables with the Bayesian network learning process to incorporate continuous variables, making full use of the original data and ensuring the rationality of discretization [19]. In this study, the HBN model exhibited higher model efficacy than the traditional logistic model, similar to the results of some previous studies. In addition, when the constructed model was applied to the subgroup population for prediction, the difference between the accuracy and the net clinical benefit of the two models widened, and the HBN model showed better robustness.

The most common nonparametric method for estimating the survival distribution is the Kaplan-Meier (K-M) estimate [44, 45]. Using the Kaplan Meier estimate to plot survival curves and the Log Rank test to evaluate survival differences between two groups is a commonly used univariate analysis method for survival data. For example, the study have compared the survival rate between patients with adenocarcinoma of the lung to receive either oral uracil tetrafur for two years or no treatment using the Kaplan Meier method and Log Rank test. This study found that the difference in overall survival between the two groups was statistically significant in favor of the uracil-tegafur group [46]. The K-M curve and Log Rank test can only be used for univariate analysis, but the Cox portational hazards(Cox) model can simultaneously analyze the impact of multivariate analysis on outcome events. In addition, Cox regression can also predict survival probability, which is the same as the role of the logistic regression prediction model in this regard [28]. Although we uses survival data, we focus on whether the patient will survive after 18 months, so we choose the logistic regression and Bayesian network prediction model. In previous studies on predictive models for subgroup analysis, most models were constructed using the whole population and validated and evaluated in both the whole and subgroup populations [47], and few models were constructed and validated using subgroup populations [48]. The model was constructed from the overall population and validated in both the overall population and subgroups to determine the ideal population for the model. xuezhi et al. used multiparametric magnetic resonance imaging (MRI) radiological signals to predict lymph node status after neoadjuvant therapy. They applied the prediction model to the T1-2 and T3-4 populations, respectively. The results showed that the overall population’ negative predictive value (NPV) was 93.7%. The NPVs of the T1-2 and T3-4 subgroups were 100 and 87.8%, respectively, which were generally consistent with the results of this study. That is, the predictive models constructed using the overall patients showed different model performances when validated in different subgroups, with lower predictive performance for advanced or high-risk patients and higher predictive performance for early-stage patients. Predictive efficiency was higher in early-stage patients. Previous studies have also concluded that prognostic models perform well in training cohorts but are less accurate in high-risk patients, younger or older patients [49]. Possible reasons are that factors such as demographic characteristics and disease characteristics are not sufficient to predict survival very accurately in advanced HER2 + patients and that the treatment modality taken by the patient is also an important influencing factor, for example, treatment with one or more drugs such as pyrrolizidine, docetaxel, trastuzumab, or drug combination with neoadjuvant therapy can effectively improve the prognosis of HER2 + patients [7]. Since HER2 + patients are relatively few, accounting for only 15–20% of breast cancer patients [4], deviations in their survival estimates may not affect the model’s overall accuracy. Nevertheless, from an individual perspective, overestimation or underestimation of survival may alter the treatment modalities and treatments adopted by patients and physicians, with serious consequences [50, 51].

From the network structure and arc strength (Supplementary Tables 1, Additional File 1), it can be seen that 17 variables have a direct impact on survival outcomes. The most influential factors are stage and distance metastasis status, severity, PR receiver, ER receiver, lymph node, tumor size, historical type, and grade. Other variables have less impact on the outcome. The HBN model has high sensitivity and specificity, especially its specificity.

There are also some limitations in this study. First, the data used in this study for both internal and external validation were from the SEER database. However, external validation in real-world data would have been more indicative of the extrapolation of the model. Secondly, the data follow-up time was short and only predicted the survival rate of breast cancer patients at one year, and it might be more meaningful to add the prediction results at three and five years. Again, there were fewer continuous variables, only age and tumor size, which could not fully reflect the advantages of the hybrid Bayesian network. Finally, in the SEER data, “none” and “unknown” are combined into one category in variables such as chemotherapy and radiotherapy, so we cannot ignore the omission.

## Conclusion

In conclusion, the hybrid Bayesian network model for breast cancer represents the interaction between disease and factors in a graphical form intuitively and reasonably and has high predictive accuracy, which can assist clinical decision-making and improve the net benefit of disease treatment.

## Electronic supplementary material

Below is the link to the electronic supplementary material.

**Fig. S1** ROC curve for internal validation of LR model. **Fig. S2** ROC curve for internal validation of HBN model. **Fig. S3** Calibration curve for internal validation of LR model. **Fig. S4** Calibration curve for internal validation of HBN model. **Fig. S5** ROC curve for external validation of LR model. **Fig. S6** ROC curve for external validation of HBN model. **Fig. S7** Calibration curve for external validation of LR model. **Fig. S8** Calibration curve for external validation of HBN model. **Fig. S9** DCA for external validation of LR model. **Fig. S10** DCA for external validation of HBN model. **Table S1** Arc between survival and other nodes

## Data Availability

The datasets generated and analyzed during the current study are available in the SEER repository, https://seer.cancer.gov/. A formal request must be made to the SEER program to access the research data, https://seerdataaccess.cancer.gov/seer-data-access.

## List of abbreviations

HBN: Hybrid Bayesian Network
LR: Logistics Regression
HER2: Human Epidermal Growth Factor Receptor-2
RF: Random Forest
ANN: Artificial Neural Network
DT: Decision Tree
SVM: Support Vector Machine
BN: Bayesian Network
SEER: The Surveillance, Epidemiology, and End Results
CGBNs: The Conditional Gaussian Bayesian network
L_DVBN: The Learning Discrete Valued Bayesian Networks
AUC: Area Under Curve
ROC: Receiver Operating Characteristic
DCA: Decision Curve Analysis
