# Supplementary material for: Prognostic models for breast cancer: based on logistics regression and Hybrid Bayesian Network
Source: BMC Med Inform Decis Mak. 2023 Jul 13;23:120. doi: 10.1186/s12911-023-02224-1 (PMC10347801; doi:10.1186/s12911-023-02224-1)
Supplement: Supplementary file 1 — Fig. S1 ROC curve for internal validation of LR model. Fig. S2 ROC curve for internal validation of HBN model. Fig. S3 Calibration curve for internal validation of LR model. Fig. S4 Calibration curve for internal validation of HBN model. Fig. S5 ROC curve for external validation of LR model. Fig. S6 ROC curve for external validation of HBN model. Fig. S7 Calibration curve for external validation of LR model. Fig. S8 Calibration curve for external validation of HBN model. Fig. S9 DCA for external validation of LR model. Fig. S10 DCA for external validation of HBN model. Table S1 Arc between survival and other nodes [file 12911_2023_2224_MOESM1_ESM.docx]

**article title**: Prognostic Models for Breast Cancer: Based on Logistics Regression and Hybrid Bayesian Network

**author names, affiliation and e-mail address:**

Fan Su^1^, Pei Liu^1^, Bowen Zhang^1^, Na Zhang^1^, Zongyu Luo^2^, Jiayin Han^1^, Jianqian Chao^1,2^

1. Department of Epidemiology and Health Statistics, School of Public Health, Southeast University, Nanjing, Jiangsu 210009; 2. Department of Medical Insurance, School of Public Health, Southeast University, Nanjing, Jiangsu 210009

Author: Fan Su, E-mail: sufan1113@126.com

Corresponding Author: Jianqian Chao, E-mail: chaoseu@163.com

**supplementary:**


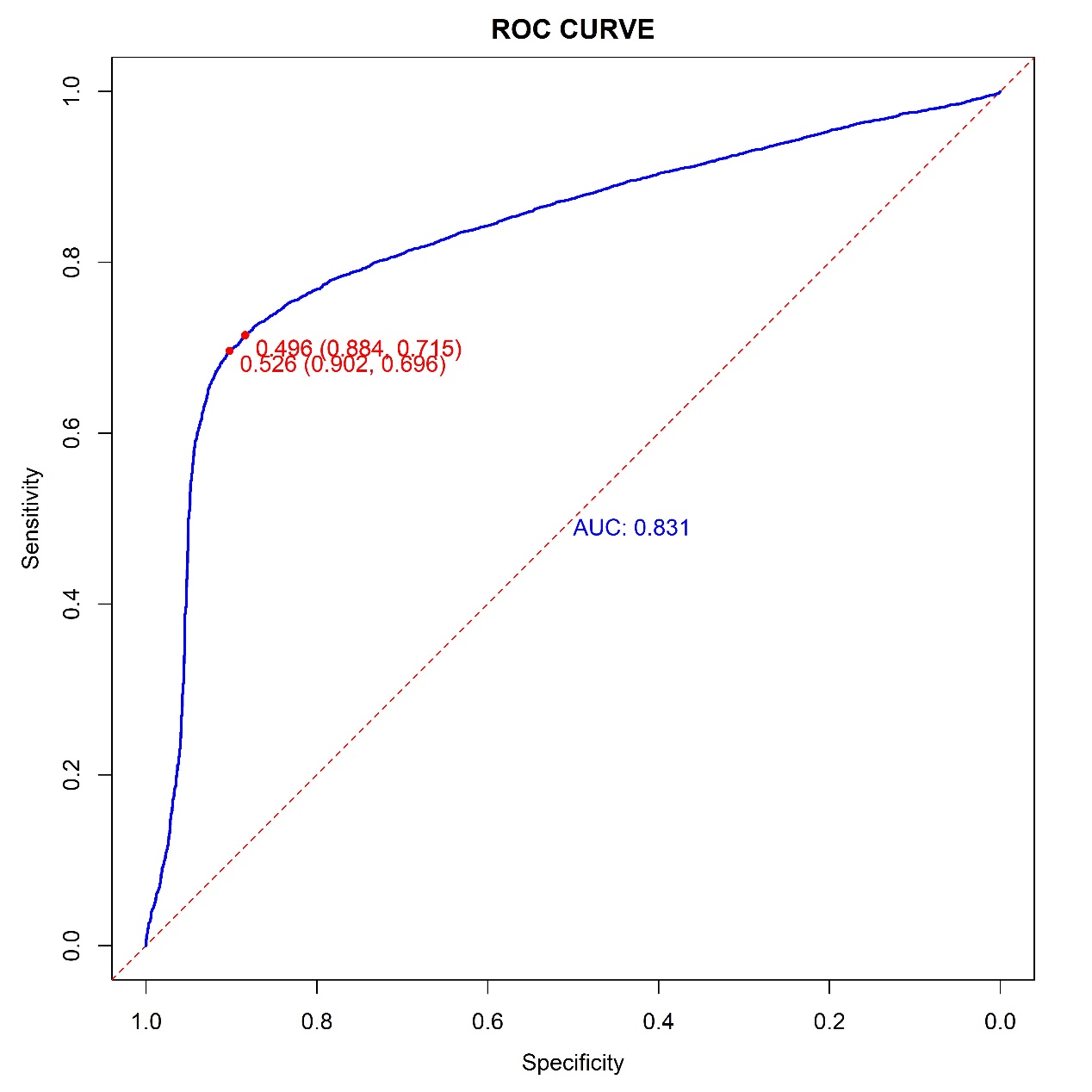


Fig. S1 ROC curve for internal validation of LR model


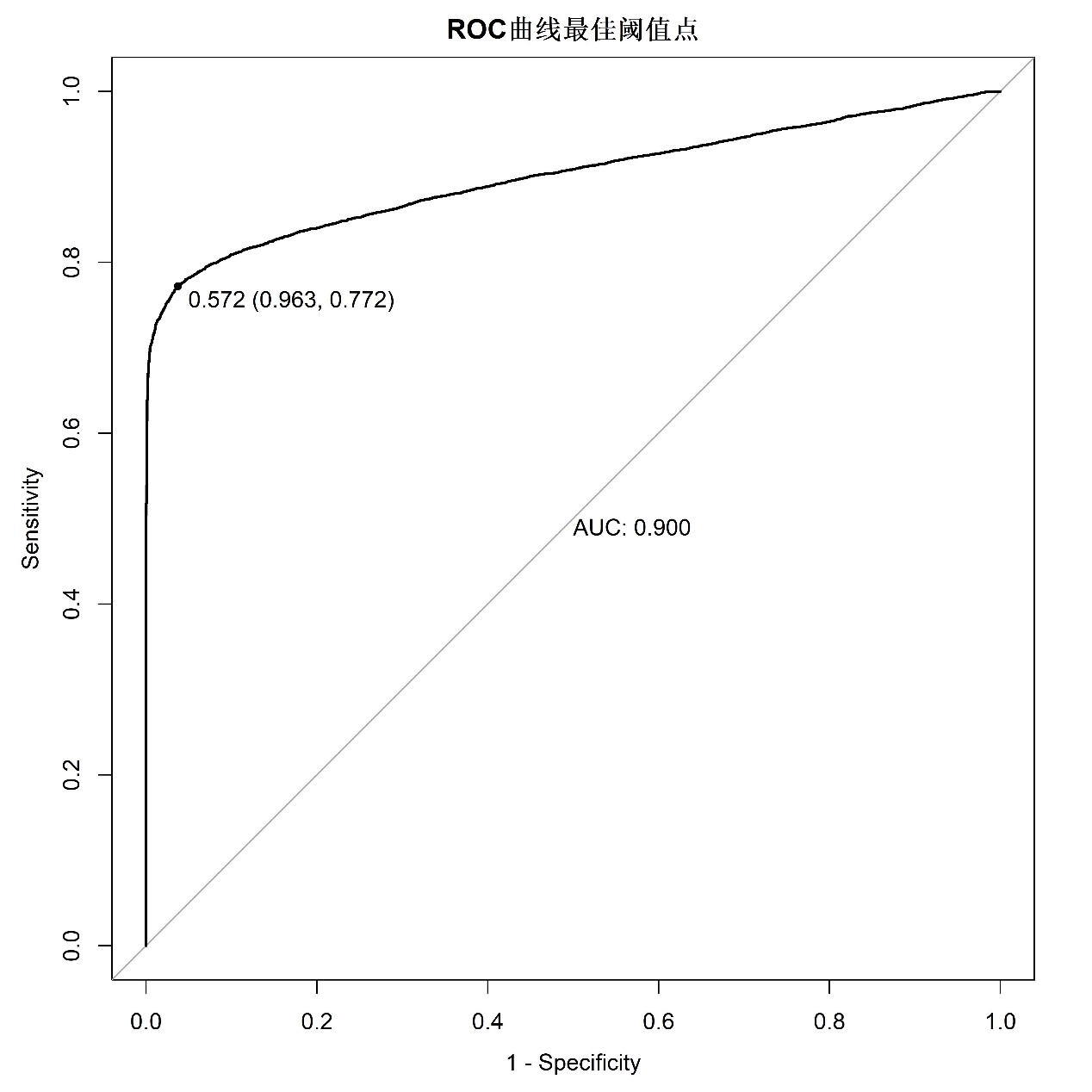


Fig. S2 ROC curve for internal validation of HBN model


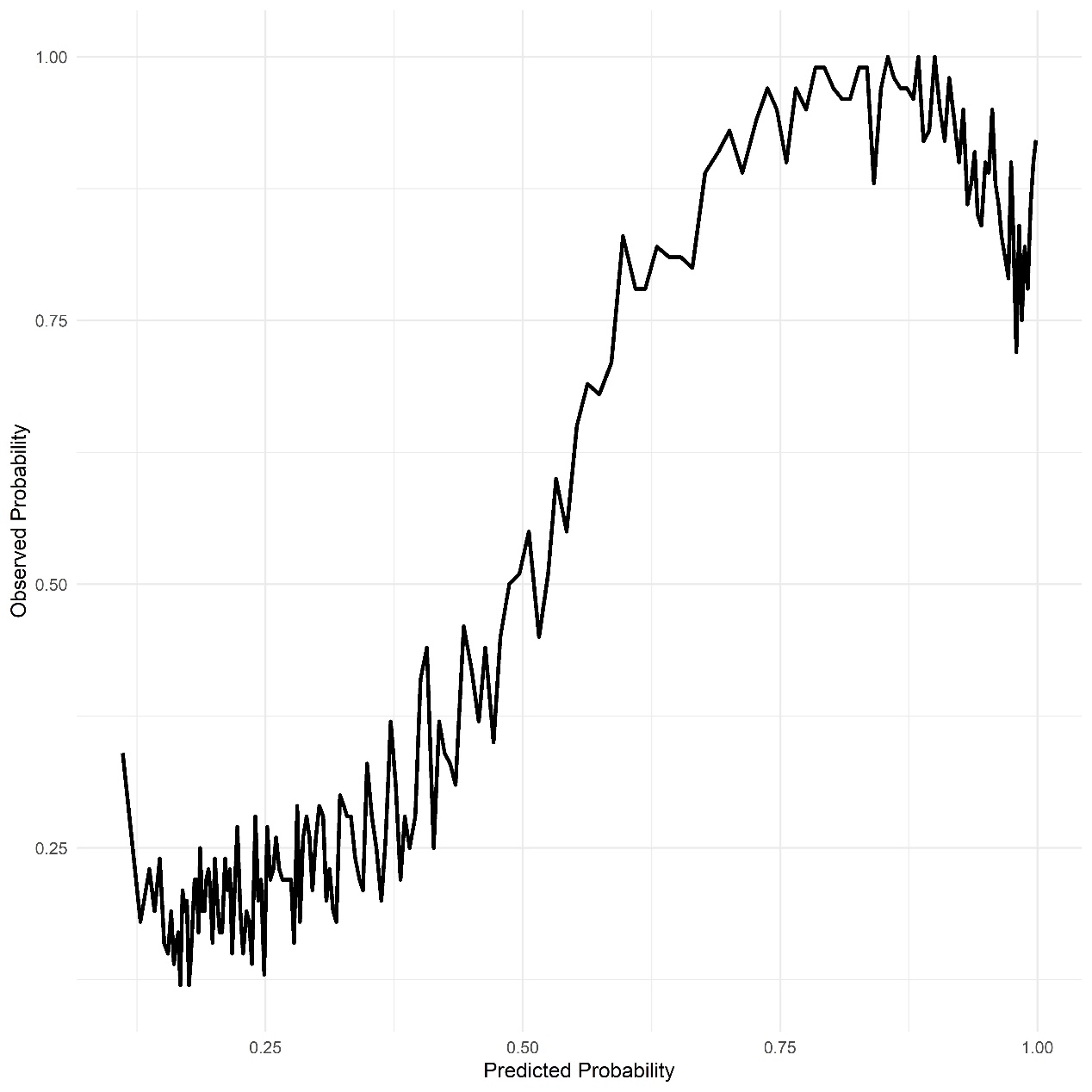


Fig. S3 Calibration curve for internal validation of LR model


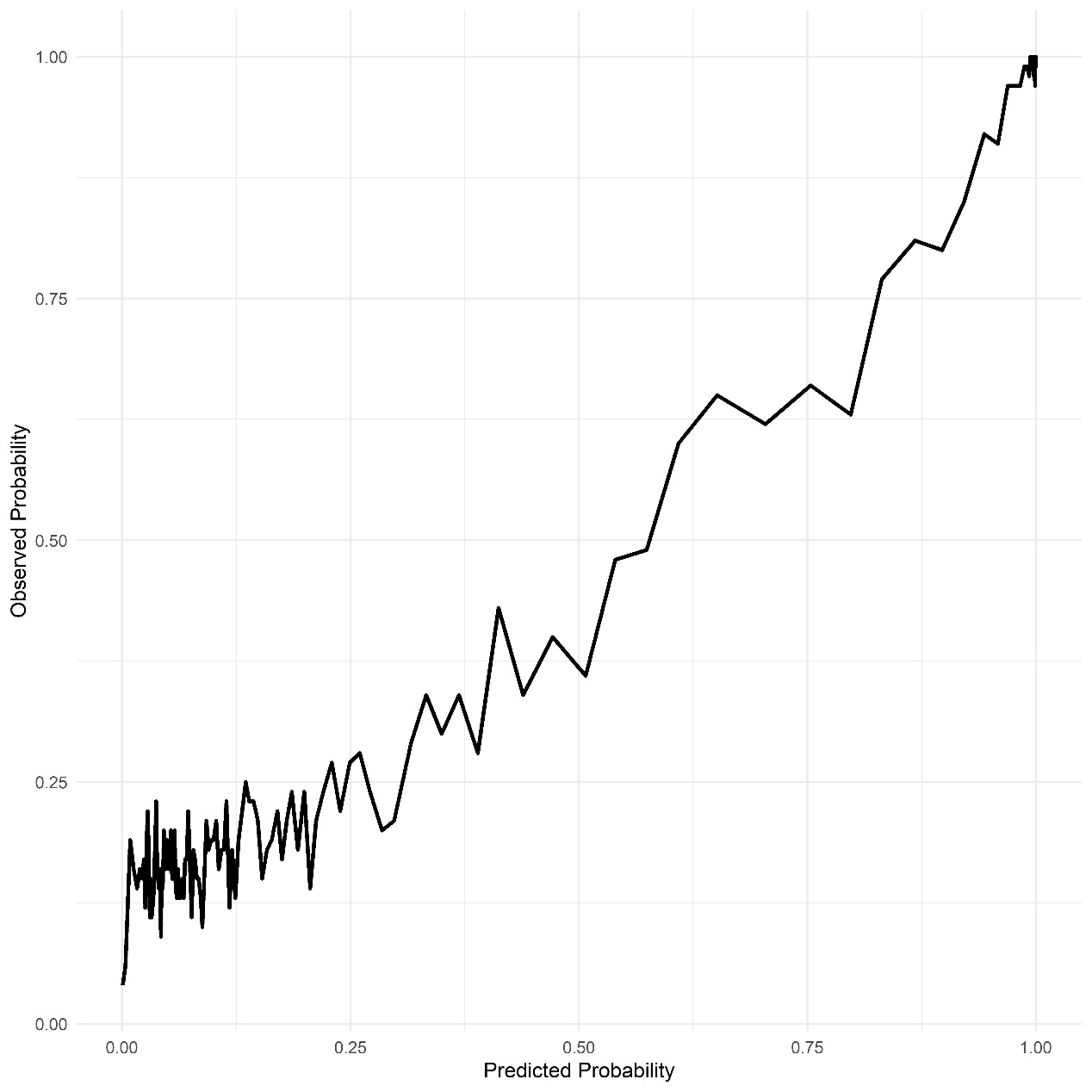


Fig. S4 Calibration curve for internal validation of HBN model


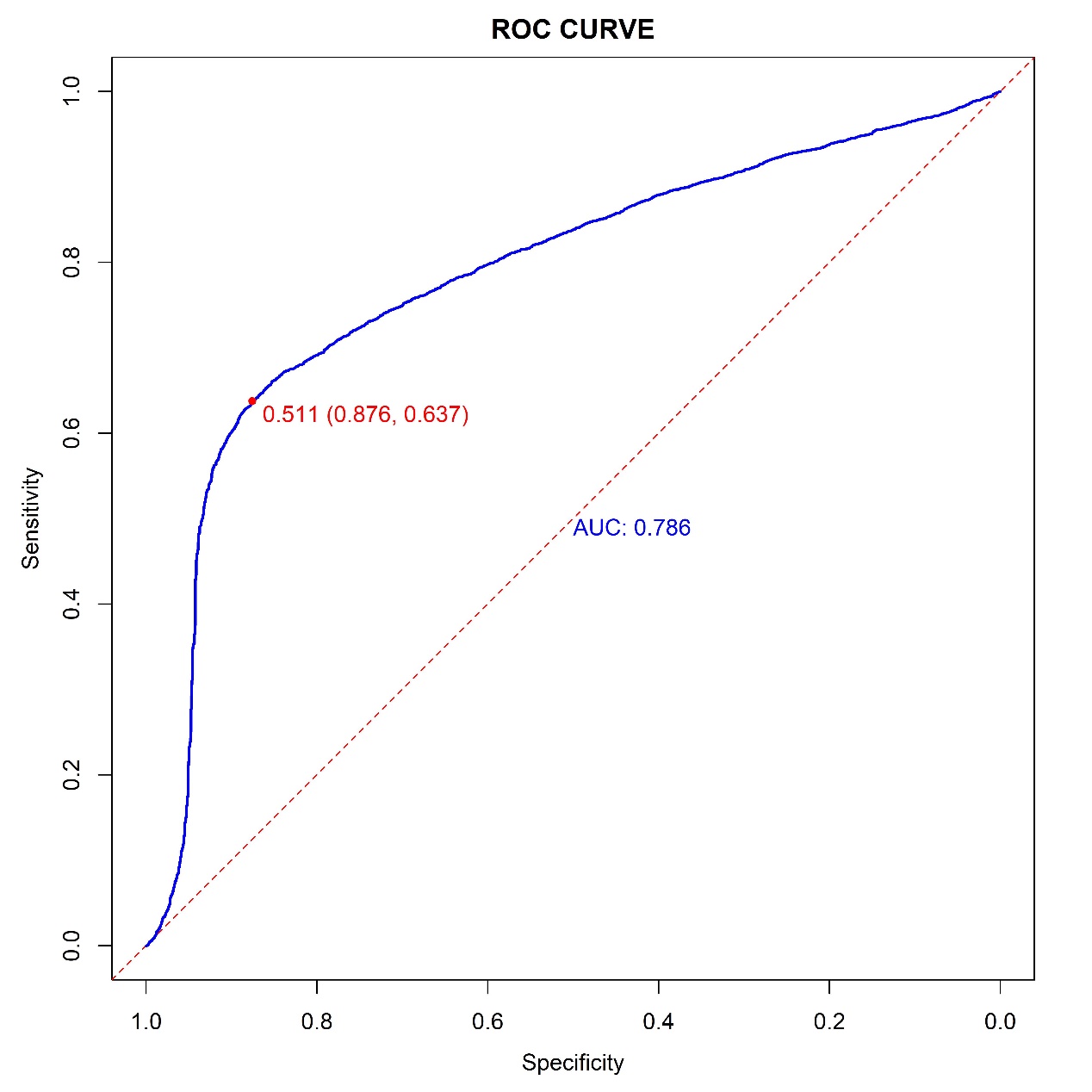


Fig. S5 ROC curve for external validation of LR model


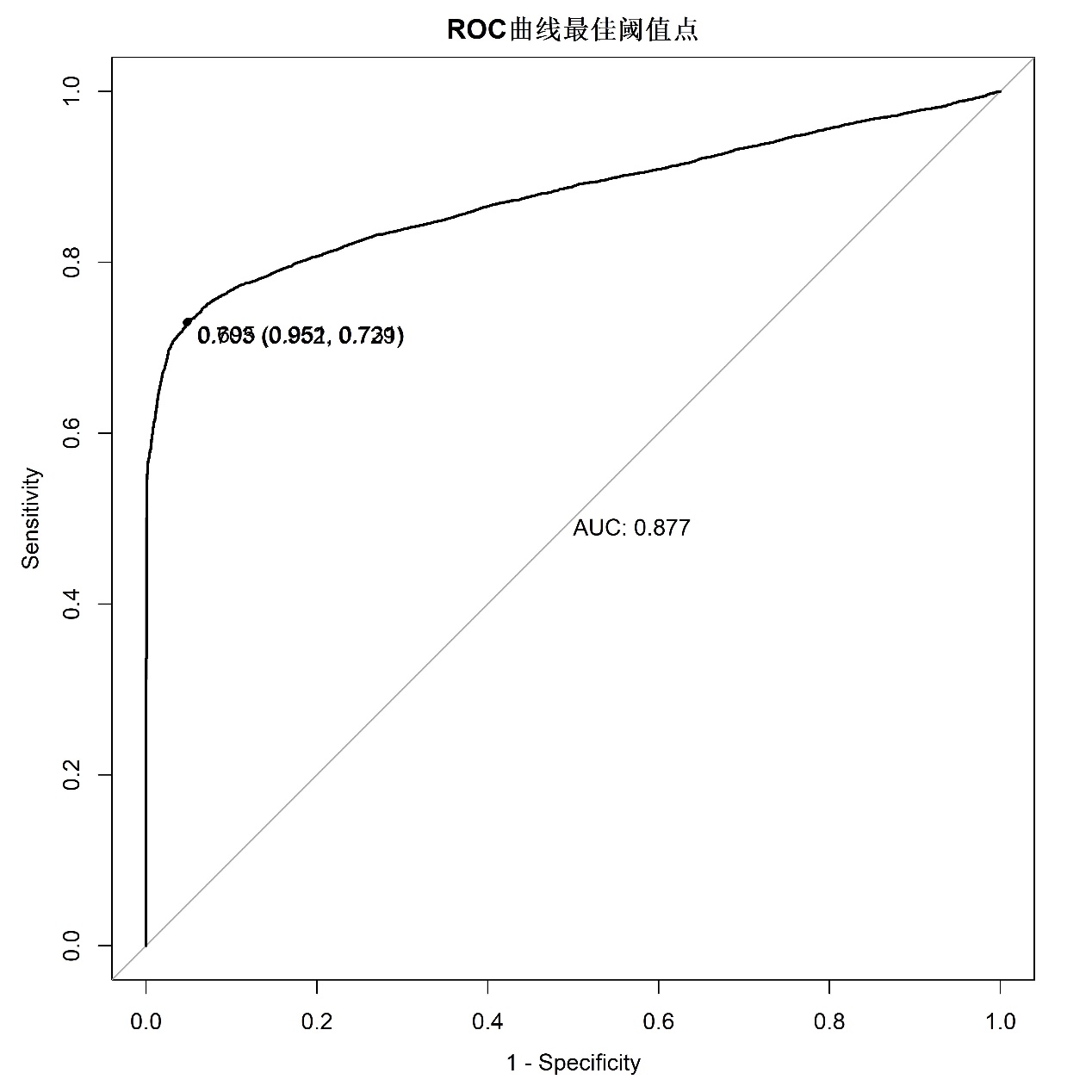


Fig. S6 ROC curve for external validation of HBN model


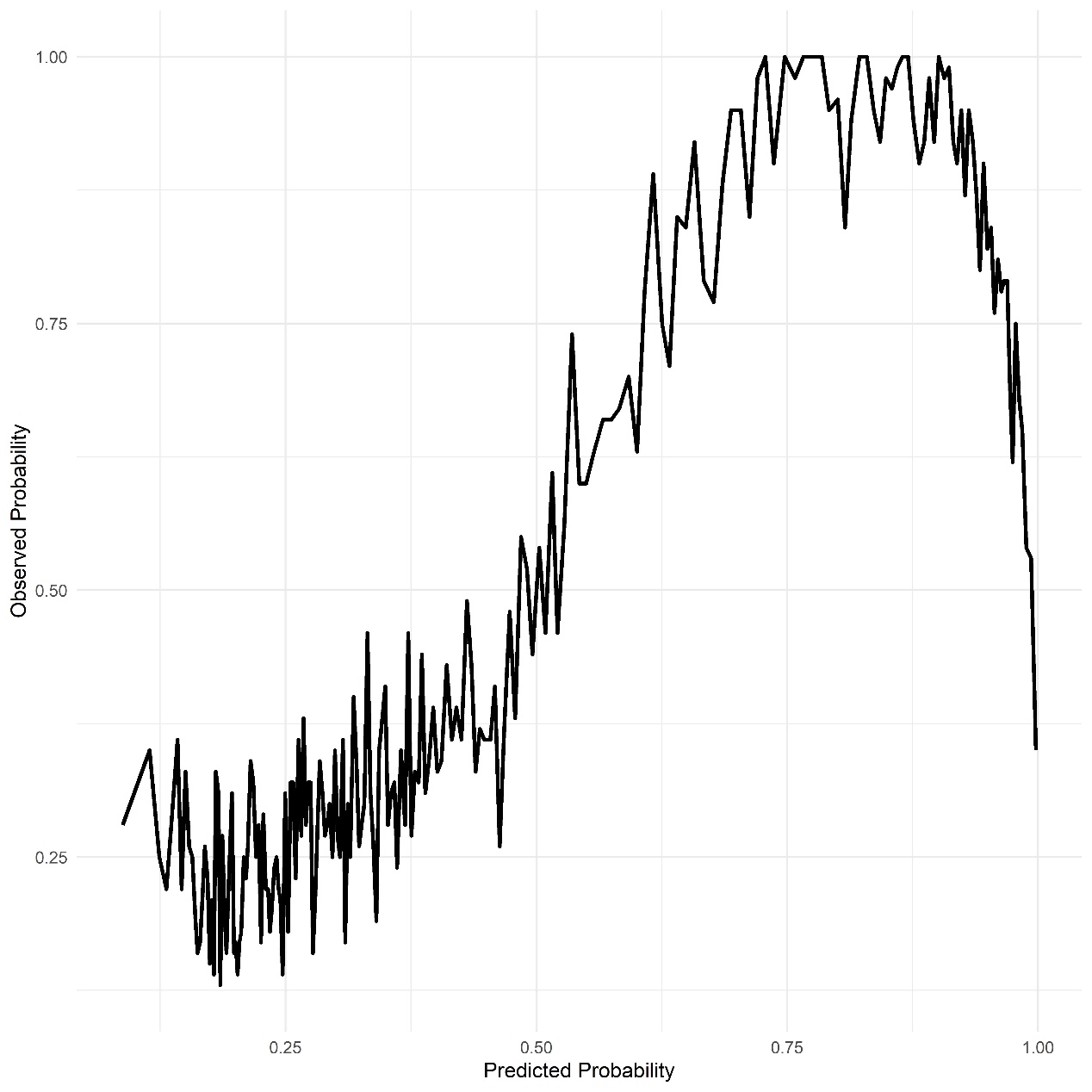


Fig. S7 Calibration curve for external validation of LR model


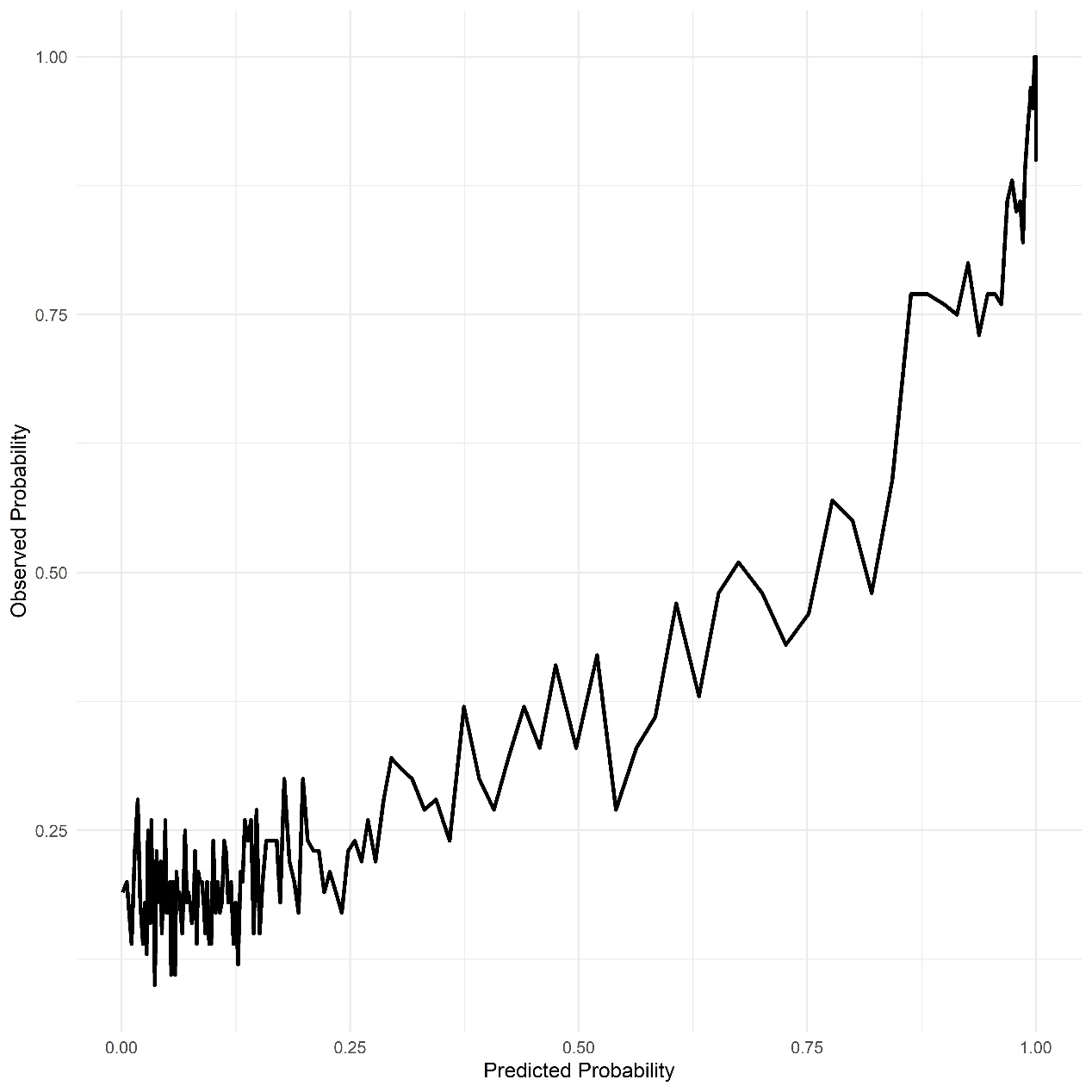


Fig. S8 Calibration curve for external validation of HBN model


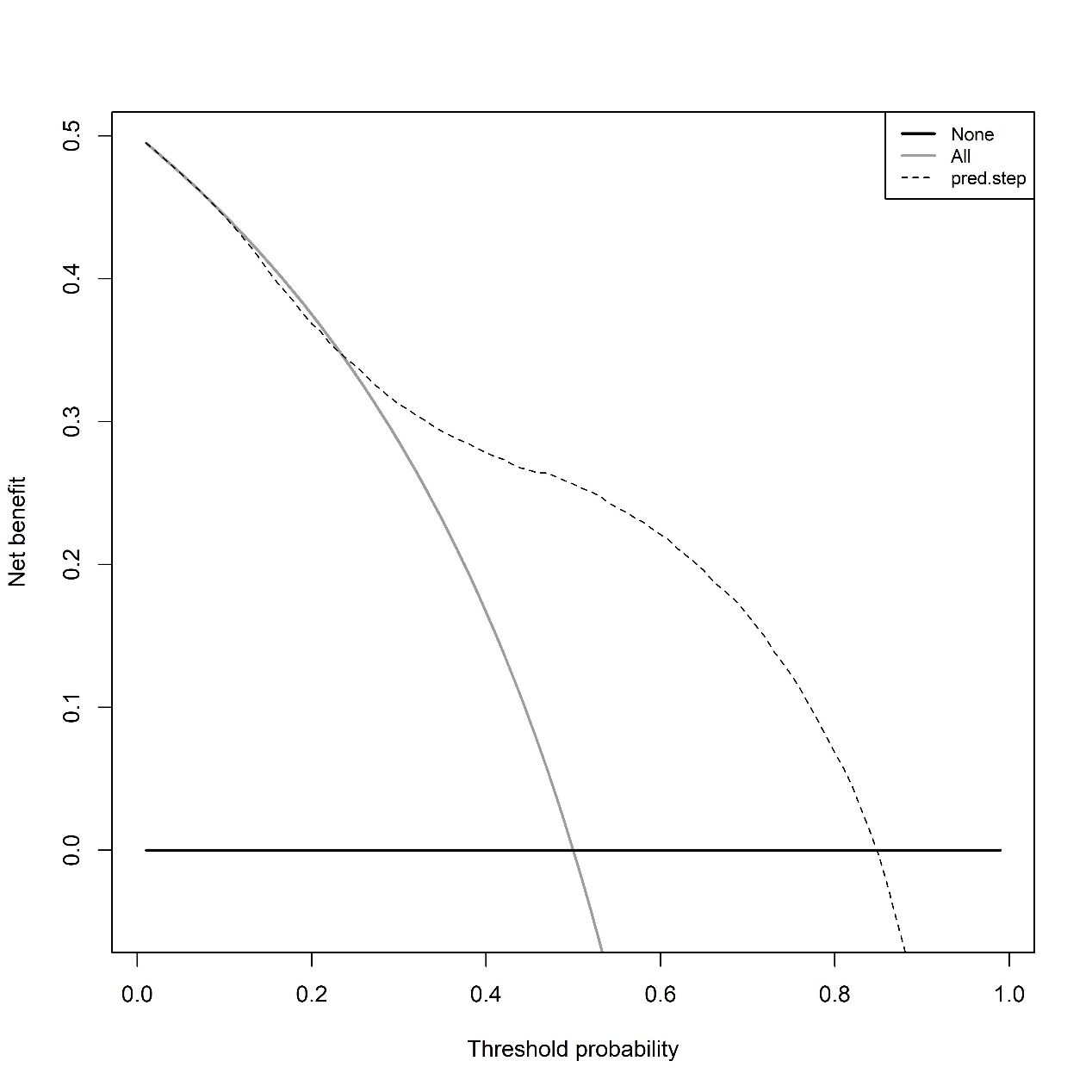


Fig. S9 DCA for external validation of LR model

†: The abscissa is the threshold probability, and the ordinate is the net benefit rate. “None” denotes that overall death occurred in no patients, with a net benefit of zero. “All” shows all patients will have overall death at a specific threshold probability. "pred. step" represents the net benefit when using the LR model as a basis for decision.


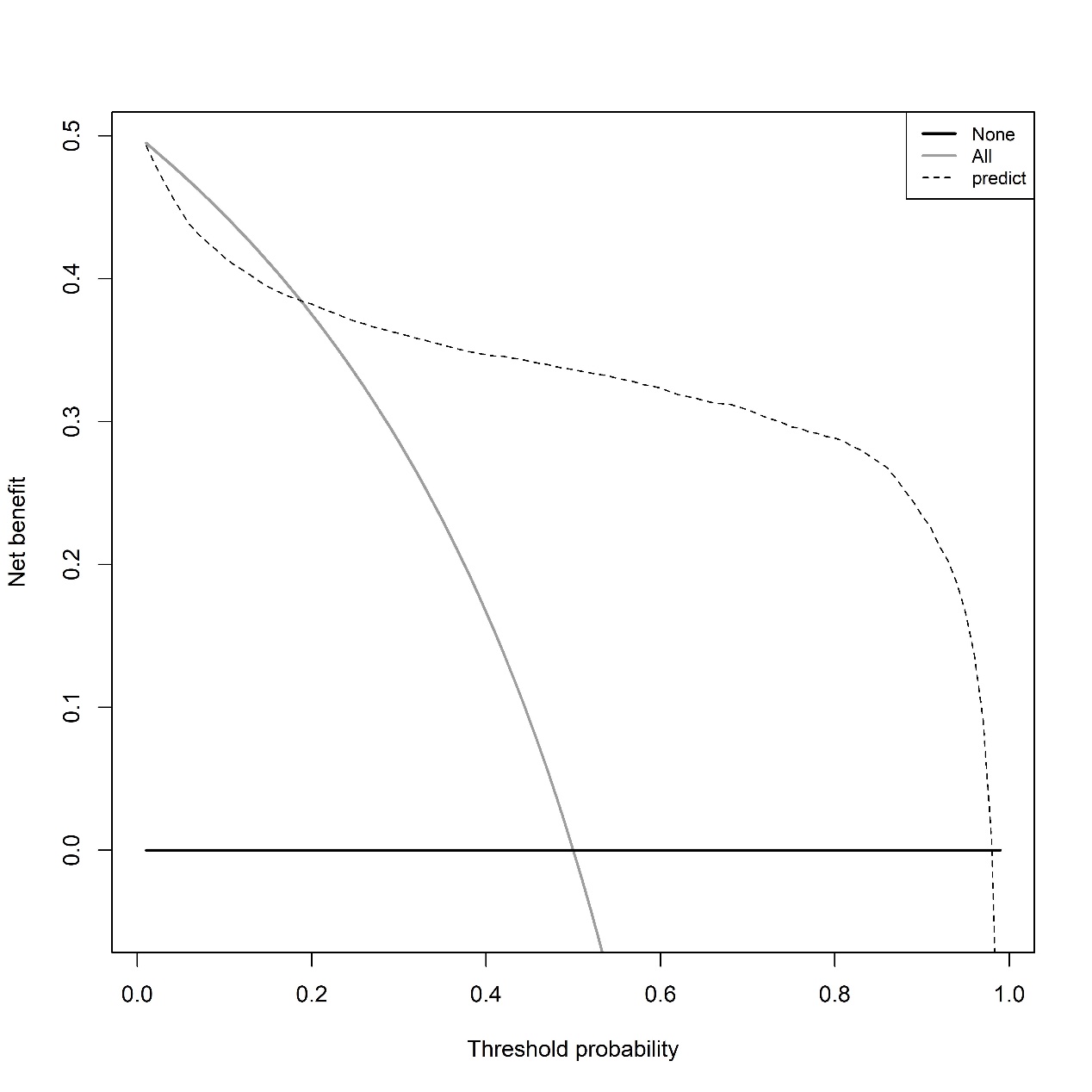


Fig. S10 DCA for external validation of HBN model

†: The abscissa is the threshold probability, and the ordinate is the net benefit rate. “None” denotes that overall death occurred in no patients, with a net benefit of zero. “All” shows all patients will have overall death at a specific threshold probability. "pred. step" represents the net benefit when using the LR model as a basis for decision.

Table S1 Arc between survival and other nodes

| from | to | strength |
| --- | --- | --- |
| Survival | Stage | -4295.36 |
| Survival | Mets | -4267.99 |
| Survival | Surgery | -2136.57 |
| Survival | PR | -2095.68 |
| Survival | ER | -2075.79 |
| Survival | Nodes | -1724.67 |
| Survival | Tumor size | -1390.76 |
| Survival | Histologic | -1217.22 |
| Survival | Grade | -1181.1 |
| Survival | Subtype | -567.36 |
| Survival | Race | -508.991 |
| Survival | Radiation | -424.389 |
| Survival | Marital | -398.838 |
| Survival | Site | -266.725 |
| Survival | age | -168.894 |
| Survival | Chemotherapy | -92.3644 |
| Survival | Laterality | -24.0699 |

Surg, Primary Site Surgery; Nodes, Lymph Node; Mets, Distant Metastasis; ER, Estrogen Receptor; PR, Progesterone Receptor; Histologic, Histologic Type; Site, Primary Site
